# Tumor necrosis factor inhibitor therapy but not standard therapy is associated with resolution of erosion in the sacroiliac joints of patients with axial spondyloarthritis

**DOI:** 10.1186/ar4548

**Published:** 2014-04-22

**Authors:** Susanne J Pedersen, Stephanie Wichuk, Praveena Chiowchanwisawakit, Robert G Lambert, Walter P Maksymowych

**Affiliations:** 1 Copenhagen Center for Arthritis Research, Center for Rheumatology and Spine Diseases, University of Copenhagen, Copenhagen, Denmark; 2 Department of Medicine, Spondyloarthritis Research Consortium of Canada Center, University of Alberta, 562 Heritage Medical Research Building, Edmonton, Alberta T6G 2S2, Canada; 3 Department of Medicine, Siriraj Hospital, Mahidol University, Bangkok, Thailand; 4 Department of Radiology and Diagnostic Imaging, Spondyloarthritis Research Consortium of Canada Center, University of Alberta, Edmonton, Alberta, Canada

## Abstract

**Introduction:**

Radiography is an unreliable and insensitive tool for the assessment of structural lesions in the sacroiliac joints (SIJ). Magnetic resonance imaging (MRI) detects a wider spectrum of structural lesions but has undergone minimal validation in prospective studies. The Spondyloarthritis Research Consortium of Canada (SPARCC) MRI Sacroiliac Joint (SIJ) Structural Score (SSS) assesses a spectrum of structural lesions (erosion, fat metaplasia, backfill, ankylosis) and its potential to discriminate between therapies requires evaluation.

**Methods:**

The SSS score assesses five consecutive coronal slices through the cartilaginous portion of the joint on T1-weighted sequences starting from the transitional slice between cartilaginous and ligamentous portions of the joint. Lesions are scored dichotomously (present/absent) in SIJ quadrants (fat metaplasia, erosion) or halves (backfill, ankylosis). Two readers independently scored 147 pairs (baseline, 2 years) of scans from a prospective cohort of patients with SpA who received either standard (*n* = 69) or tumor necrosis factor alpha (TNFα) inhibitor (*n* = 78) therapy. Smallest detectable change (SDC) was calculated using analysis of variance (ANOVA), discrimination was assessed using Guyatt’s effect size, and treatment group differences were assessed using t-tests and the Mann–Whitney test. We identified baseline demographic and structural damage variables associated with change in SSS score by univariate analysis and analyzed the effect of treatment by multivariate stepwise regression adjusted for severity of baseline structural damage and demographic variables.

**Results:**

A significant increase in mean SSS score for fat metaplasia (*P* = 0.017) and decrease in mean SSS score for erosion (*P* = 0.017) was noted in anti-TNFα treated patients compared to those on standard therapy. Effect size for this change in SSS fat metaplasia and erosion score was moderate (0.5 and 0.6, respectively). Treatment and baseline SSS score for erosion were independently associated with change in SSS erosion score (β = 1.75, *P* = 0.003 and β = 0.40, *P* < 0.0001, respectively). Change in ASDAS (β = −0.46, *P* = 0.006), SPARCC MRI SIJ inflammation (β = −0.077, *P* = 0.019), and baseline SSS score for fat metaplasia (β = 0.085, *P* = 0.034) were independently associated with new fat metaplasia.

**Conclusion:**

The SPARCC SSS method for assessment of structural lesions has discriminative capacity in demonstrating significantly greater reduction in erosion and new fat metaplasia in patients receiving anti-TNFα therapy.

## Introduction

Radiography of the sacroiliac joint (SIJ) in axial spondyloarthritis (SpA) is a valuable diagnostic tool but is unreliable and unresponsive for assessment of disease-modifying treatment effects. There is therefore an unmet need for imaging tools to assess the potential disease-modifying effects of therapeutic agents early in SpA when disease is still confined to the SIJ. Magnetic resonance imaging (MRI) represents a substantial advance in the field due to its ability to visualize inflammation in soft tissue as well as subchondral bone. This is evident on fat-suppressed sequences such as short tau inversion recovery. Recent MRI data also show that resolution of inflammation may be associated with the development of fat metaplasia on the T1-weighted spin echo (T1WSE) sequence [1-3]. Fat metaplasia is not observed on radiography and the histopathology of this lesion is unknown, but it is frequently observed in SIJs and at spinal locations that are also typical for inflammation; that is, vertebral corners, adjacent to vertebral endplates, facet and costo-vertebral joints [4]. We have previously hypothesized that resolution of inflammation in erosions is followed by development of a new tissue, which on T1WSE MRI has the same signal intensity as fat metaplasia [5]. We have called this type of lesion backfill due to its appearance in the cavity of the erosion, whereas the term fat metaplasia is used when this lesion is located in the bone marrow.

There has been limited assessment of MRI-based scores for structural lesions in the SIJ in clinical trials, and little is known regarding the impact of different therapies. One study has reported that scoring fat metaplasia may discriminate between therapies in a time frame as short as 6 months [3]. However, the significance of this for structural damage progression is unclear. The Spondyloarthritis Research Consortium of Canada (SPARCC) MRI Sacroiliac Joint Structural Score (SSS) is a new scoring instrument that assesses a broader spectrum of structural lesions in the SIJ, which include erosion, fat metaplasia, backfill, and ankylosis [6]. Because of the increasing focus on effective treatment intervention in early axial SpA, there is a need to validate this new scoring instrument for its potential to discriminate between therapies. The aim of this study was to investigate whether there are differences in structural progression on MRI in patients with axial SpA treated with or without tumor necrosis factor-alpha (TNFα) inhibitor when assessed using the SPARCC MRI SSS.

## Methods

### Patients

We assessed scans from patients with available baseline and 2-year MRI scans and meeting the modified New York criteria [7] recruited in a consecutive manner to a prospective cohort. Patients are recruited from community-based clinical practice and academic-based outpatient facilities in the city of Edmonton irrespective of what treatment they have received. Baseline and 2-year MRI scans were available for 147 patients with axial SpA meeting the modified New York criteria [7], who had been evaluated systematically according to a standardized protocol. Of these, 68 patients received standard therapies (nonsteroidal anti-inflammatory agents and/or physiotherapy) and 79 patients initiated TNFα inhibitor therapy. In addition to demographic variables, the Assessments in Spondyloarthritis International Society core set is used to assess signs and symptoms of disease activity [8], and C-reactive protein (CRP) (mg/L) and the SPARCC MRI SIJ inflammation scores are used to objectively assess degree of inflammation [9]. Assessments are conducted at baseline, at 3 to 6 months for patients starting TNFα inhibitor, and annually for all patients as described previously [10]. SPARCC MRI SIJ inflammation scores are recorded for each patient in the cohort by a reader unconnected with this study.

### Study approval

The study received ethical approval from the Health Research Ethics board of the University of Alberta and was performed in accordance with the Helsinki Declaration. Written informed consent was obtained from all study participants before inclusion into the observational cohort.

### Magnetic resonance imaging protocol

Scans were semi-coronal T1WSE sequences of the SIJs. The scan parameters were as follows: 15 to 19 slices, 4 mm slice thickness, 0.4 mm interslice gap, field of view 280 to 300 mm, repetition time 423 to 450 milliseconds, echo time 12 to 13 milliseconds, echo train length 3, and matrix 512 × 256 pixels. Although scans from all patients included the short tau inversion recovery sequence, these scans were deleted from the set of scans included in this validation process to avoid simultaneously eliciting information on inflammation provided by this sequence.

### Structural lesion definitions

We adopted the following standardized definitions of structural lesions of the SIJ on MRI, which were developed by the Canada–Denmark MRI Working Group [4] and which were extended in a subsequent report to include backfill [5].

Fat metaplasia is defined as an increased signal on T1WSE. The reference for normal bone marrow signal is the marrow signal in the center of the sacrum at the corresponding craniocaudal level. In order to be scored in the SSS method, the lesion has to demonstrate a homogeneous bright signal that is at least 1 cm in depth from the joint surface.

An erosion is defined as the full-thickness loss of the dark appearance of either the iliac or sacral cortical bone *at its anticipated location* and loss of the normal bright appearance of adjacent bone marrow on T1WSE.

Backfill is defined as complete loss of iliac or sacral cortical bone *at its anticipated location* and an increased signal on T1WSE that is clearly demarcated from adjacent normal marrow by irregular dark signal reflecting sclerosis.

Finally, ankylosis is defined as a bone marrow signal on T1WSE extending between the sacral and iliac bone marrow.

Examples of structural lesions together with a module describing the SSS method and a reference image set based on Digital Imaging and Communications in Medicine images are available online [11]. This training module also includes a schematic of the SIJ for direct electronic data entry online and raw scores from two reader pairs who achieved the highest reliability in the validation exercises (*vide infra*) to facilitate calibration of nonexpert readers. Bone sclerosis and abnormalities of the synovial cavity are not addressed in the SSS method because of poor reproducibility in previous reading exercises.

### Scoring methodology

The SPARCC SSS method incorporates key scoring principles from the SPARCC SIJ inflammation score, which is based on assessment of consecutive slices through the SIJ, division of each SIJ into quadrants, and dichotomous (present/absent) scoring of lesions in each quadrant. Evaluation of structural lesions in the SIJ using the SSS method is conducted using T1WSE scans and proceeds sequentially in the following steps.

First, the transitional slice is identified by scrolling through the Digital Imaging and Communications in Medicine images from anterior to posterior semi-coronal slices through the joint. The transitional slice is defined as the first slice in the cartilaginous portion that has a visible portion of the ligamentous joint when viewed from anterior to posterior.

All time points are then anatomically matched according to the transitional semi-coronal SIJ slice. The link function on Digital Imaging and Communications in Medicine software allows simultaneous scrolling of anatomically matched images from the transitional slice anteriorly, thereby facilitating detection of change in lesions between time points.

Finally, five consecutive semi-coronal slices are assessed starting from the transitional slice and scrolling anteriorly. The SIJ cavity together with adjacent bone marrow should still be clearly visible at the most anterior slice.

The presence/absence of lesions is scored in SIJ quadrants (fat, erosion) or halves (backfill, ankylosis) using a direct online data-entry system based on a schematic of the SIJ. Scoring ranges are: fat metaplasia (0 to 40), erosion (0 to 40), backfill (0 to 20), ankylosis (0 to 20).

### Reading exercises

Reads were conducted blinded to patient demographics and treatment. We first conducted a calibration exercise of 20 cases randomly selected from the cohort with baseline and 2-year scans that were scored by two readers blinded to time point. In the primary exercise, two readers independently scored the 147 cases with baseline and 2-year scans blinded to time point. Data were directly entered online into a web-based scoring system that is illustrated as a schematic with each SIJ divided into quadrants.

### Statistical analysis

We used descriptive statistics to compare the clinical characteristics and the number (percentage) of patients with any change and the mean (standard deviation) change in each of the four structural lesion scores for the two treatment groups. Analyses were performed using the mean scores of the two readers. Comparisons of proportions of patients demonstrating any change in SSS were conducted using Fisher’s exact test. Treatment group differences were assessed using cumulative probability plots, unpaired *t* tests, and the Mann–Whitney test for nonparametric data. Correlations were analyzed using Spearman’s rho between: change in objective (CRP, SPARCC MRI SIJ inflammation score) and other (Ankylosing Spondylitis Disease Activity Score (ASDAS)) measures of inflammation; change in MRI SSS for fat metaplasia, erosion, and backfill; and change in MRI SSS for ankylosis.

If treatment group differences for change in specific structural lesion scores were significant in group analyses, we explored the potential impact of baseline differences between treatment groups on change in MRI SSS by analyzing variables related to demographics (gender, B27 status) and disease severity (SSS for erosion, fat metaplasia, backfill, ankylosis) using univariate regression, with a significant interaction defined as *P* ≤ 0.10. We then analyzed interaction effects between treatment and these variables.

The effect of treatment on change in SSS was further analyzed in multivariate stepwise regression analyses that included the following variables: age, sex, symptom duration, baseline and 2-year change in ASDAS, baseline and 2-year change in CRP, baseline and 2-year change in SPARCC SIJ inflammation score, and baseline SSS for erosion, fat metaplasia, backfill, and ankylosis. Significant interactions were further analyzed by including the interaction terms in multivariate stepwise regression analyses.

The smallest detectable change (SDC) was calculated using the Bland-Altman 80% levels of agreement and expressed as an absolute value and as a percentage of the maximum score [12]. The SDC provides an absolute measure of agreement, which can be used as a guideline for the clinicians and applied clinically for assessing real change beyond measurement error at the individual patient level. Discrimination was assessed using Guyatt’s effect size, which was calculated by dividing the mean of the change scores in the TNFα inhibitor group by the standard deviation of the change scores in the standard therapy group for each of the structural lesions. Effect sizes of at least 0.2, 0.5, and 0.8 are considered small, moderate, and large, respectively.

## Results

### Baseline characteristics

Demographic and disease characteristics at baseline showed significantly more active disease (Bath Ankylosing Spondylitis Disease Activity Index, total back pain, nocturnal back pain, patient global, ASDAS, CRP) (*P* <0.0001 for all variables) in patients who received TNFα inhibitor therapy (Table 1). Functional severity (Bath Ankylosing Spondylitis Functional Index [13]) and radiographic severity (modified Stoke Ankylosing Spondylitis Spine Score [14]) were also worse in those who received TNFα inhibitor therapy (*P* < 0.0001 for both variables). Significantly more ankylosis was recorded in the SIJ on the baseline MRI scan in patients who received TNFα inhibitor therapy (*P* = 0.02) (Table 2).

**Table 1:** Baseline demographics and disease status in 147 patients with axial spondyloarthritis receiving either standard (NSAID and/or physiotherapy) or TNFα inhibitor therapy

	**Standard therapy (*n *** **= 68)**	**TNFα inhibitor (*n *** **= 79)**
Age	40.44 (12.86)	40.29 (10.70)
Males No (%)	47 (69%)	64 (81%)
Symptom duration	16.12 (10.62)	16.94 (9.64)
BASDAI	3.71 (2.24)	5.99 (2.12)
Total back pain	3.69 (2.67)	5.75 (2.57)
Nocturnal back pain	4.03 (2.75)	6.05 (2.60)
Patient global score	3.85 (2.57)	5.91 (2.84)
BASFI	2.36 (2.56)	4.83 (2.71)
HLA B27^a^	55 (87%)	58 (85%)
C-reactive protein	6.83 (9.53)	18.37 (18.84)
ASDAS	2.29 (1.02)	3.55 (1.10)
mSASSS	12.32 (18.15)	17.47 (19.08)

*ASDAS,* Ankylosing Spondylitis Disease Activity Score; *BASDAI*, Bath Ankylosing Spondylitis Disease Activity Index [15]; *BASFI*, Bath Ankylosing Spondylitis Functional Index [13]; *HLA*, human leukocyte antigen; *mSASSS*, modified Stoke Ankylosing Spondylitis Spine Score [14]; NSAID, nonsteroidal anti-inflammatory drug; TNFα, tumor necrosis factor alpha. Values represent mean (standard deviation) or number (percentage). ^a^Missing values in 5 and 11 patients of the standard therapy and TNFα inhibitor groups, respectively.

**Table 2:** Detection of change in SPARCC MRI SSS and discrimination of the SSS in 147 patients with axial spondyloarthritis receiving either standard or TNFα inhibitor therapy

	**SDC (% maximum range)**	**Mean (SD) baseline score**		**P value**	**Mean (SD) change score**		**Guyatt’s Effect size**	***P*** **value**
		**Anti-TNFα**^**a**^	**Standard**^**b**^		**Anti-TNFα**^**a**^	**Standard**^**b**^		
Fat metaplasia	2.2 (5.5%)	3.6 (5.1)	4.0 (6.6)	NS	0.7 (2.2)	0.2 (1.5)	0.5	0.017
Backfill	2.8 (14%)	3.7 (4.5)	2.2 (3.1)	0.08	0.1 (2.9)	0.4 (1.9)	0.03	NS
Erosion	3.05 (7.6%)	2.7 (3.2)	3.6 (4.4)	NS	−1.5 (2.8)	−0.5 (2.7)	0.6	0.017
Ankylosis	1.39 (7%)	7.3 (8.5)	4.1 (7.0)	0.02	0.4 (1.6)	0.3 (1.2)	0.3	NS

*MRI*, magnetic resonance imaging; NS, not significant; *SD*, standard deviation; *SDC*, smallest detectable change; *SPARCC*, Spondyloarthritis Research Consortium of Canada; *SSS*, Sacroiliac Joint Structural Score; *TNF*α, tumor necrosis factor alpha. ^a^Patients receiving TNFα inhibitor therapy (*n* = 79). ^b^Patients receiving standard therapy (nonsteroidal anti-inflammatory drug and/or physiotherapy, *n* = 68).

### Detection of change in SPARCC MRI SSS

**Figure 1:**
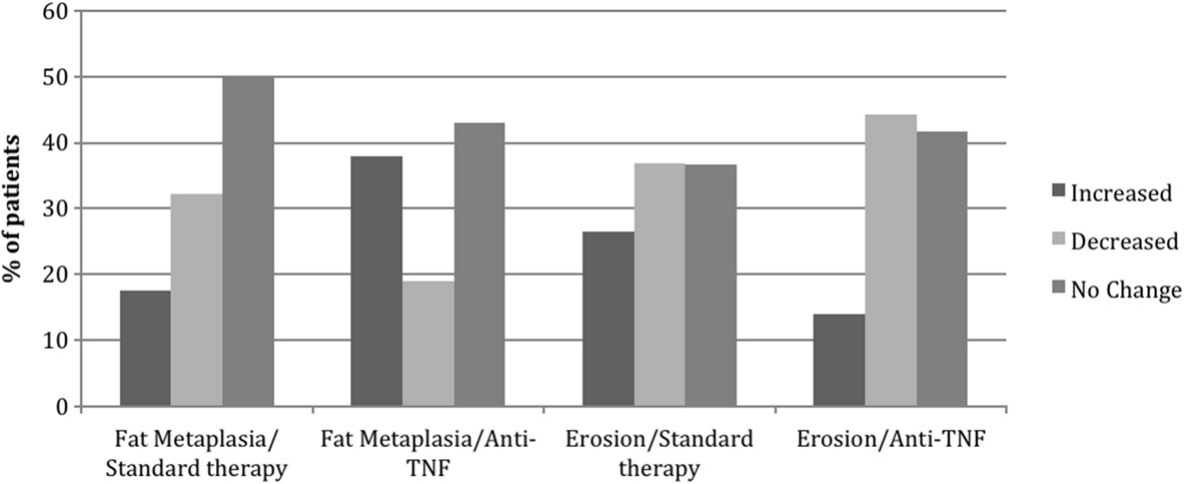
**Patients with axial spondyloarthritis demonstrating any change (increase or decrease) in Sacroiliac Joint Structural Scores for fat metaplasia or erosion.** Percentage of patients with axial spondyloarthritis who demonstrate any change (increase or decrease) in Spondyloarthritis Research Consortium of Canada magnetic resonance imaging Sacroiliac Joint Structural Scores for fat metaplasia or erosion after 2 years on either standard therapy (nonsteroidal anti-inflammatory drug and/or physiotherapy, n = 68) or tumor necrosis factor alpha (TNFα) inhibitor therapy (n = 79).

**Figure 2:**
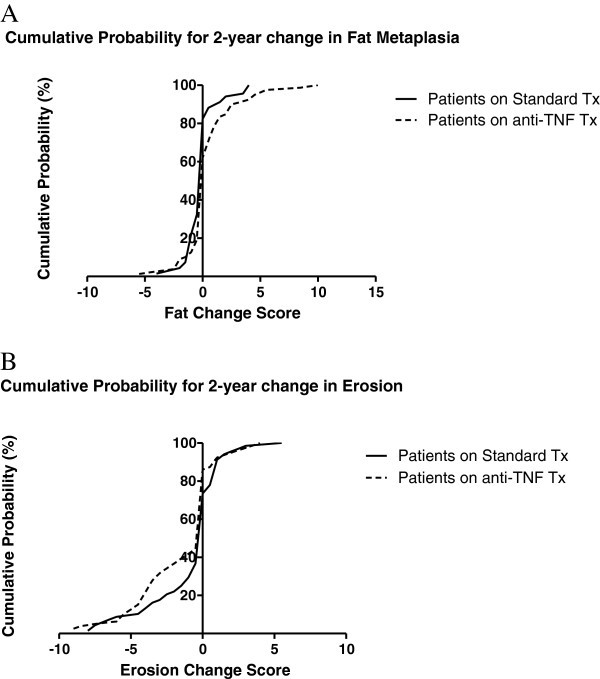
**Two-year change in mean Sacroiliac Joint Structural Scores for fat metaplasia and erosion in patients with axial spondyloarthritis.** Cumulative probability plots illustrating 2-year change in mean Spondyloarthritis Research Consortium of Canada magnetic resonance imaging Sacroiliac Joint Structural Scores for **(A)** fat metaplasia and **(B)** erosion in patients with axial spondyloarthritis on standard therapy (nonsteroidal anti-inflammatory drug and/or physiotherapy, n = 68) and tumor necrosis factor alpha (TNFα) inhibitor (n = 79) therapy. Tx, therapy.

The SDC was comparable for structural lesion scores at 5 to 7% of the scoring range although higher for backfill at 14% of the scoring range (Table 2). The number (percentage) of patients with change > SDC for the standard therapy and TNFα inhibitor groups was five (7.4%) and 15 (19%) patients for fat metaplasia, eight (11.8%) and 15 (19%) patients for Backfill, 12 (17.6%) and 24 (30.4%) patients for erosion, and seven (10.3%) and 11 (13.9%) patients for Ankylosis, respectively. A significant increase in mean SSS for fat metaplasia (*P* = 0.017) and a decrease in mean SSS for erosion (*P* = 0.017) was noted in TNFα inhibitor-treated patients compared with those on standard therapy. The effect size for the change in SSS fat metaplasia and erosion score in the TNFα inhibitor versus standard therapy groups was moderate (0.5 and 0.6, respectively). There was a significantly higher percentage of patients who developed new fat metaplasia on TNFα inhibitor therapy (38.0%) versus those who received standard therapy (17.6%) (*P* = 0.01), while more patients on standard therapy had a decrease in fat metaplasia compared with those on TNFα inhibitor therapy (32.3% vs. 19.0%, respectively) (Figure 1). Significantly more patients on standard therapy developed new erosion compared with those on TNFα inhibitor therapy (26.5% vs. 13.9%, respectively, *P* = 0.037) (Figure 1). These differences between treatment groups are further evident on the cumulative probability plots (Figure 2).

### Correlations between change in SPARCC MRI SSS

Significant correlation was noted between 2-year change in SPARCC MRI SIJ inflammation score and change in SSS for erosion in patients in both treatment groups (*r* = 0.29 (*P* = 0.032) and *r* = 0.49 (*P* < 0.0001) for standard therapy and TNFα inhibitor groups, respectively) (Table 3). There was a significant inverse correlation between change in SPARCC MRI SIJ inflammation score and change in CRP with change in SSS for fat metaplasia in patients on TNFα inhibitor therapy (*r* = −0.40 (*P* = 0.001) and *r* = −0.27 (*P* = 0.023), respectively). There was no significant correlation between change in CRP and change in SSS for fat metaplasia in the standard therapy group. The correlation between SPARCC MRI SIJ inflammation score and fat metaplasia was significant in the standard therapy group (*r* = −0.28 (*P* = 0.041)) but this was weaker compared with the TNFα inhibitor group. Decreased SSS for erosion correlated strongly with increased SSS for fat metaplasia in TNFα inhibitor-treated patients (*r* = −0.54 (*P* < 0.0001)) but not in standard therapy-treated patients. Decreased SSS for erosion correlated significantly with increased SSS for ankylosis in both treatment groups (*r* = −0.33 (*P* = 0.0058) and *r* = −0.33 (*P* = 0.0027) for standard therapy and TNFα inhibitor groups, respectively). Increase in SSS for fat metaplasia correlated significantly with increased SSS for ankylosis in TNFα inhibitor-treated patients (*r* = 0.35 (*P* = 0.0018)), but not in those receiving standard therapy.

**Table 3:** Correlation matrix demonstrating associations between changes in inflammatory parameters and structural lesions on MRI in patients with axial spondyloarthritis receiving either standard or TNFα inhibitor therapy

**TNFα inhibitor therapy (*n *** **= 79)**	**Standard therapy (*n *** **= 68)**						
	**CRP**	**ASDAS**	**SPARCC MRI SIJ inflammation score**	**SSS for fat metaplasia**	**SSS for erosion**	**SSS for backfill**	**SSS for ankylosis**
CRP		0.27, *P* = 0.05	0.19, *P* = 0.NS	0.13, *P* = 0.NS	0.16, *P* = NS	−0.03, *P* = NS	−0.19, *P* = NS
ASDAS	0.27, *P* = 0.042		0.15, *P* = NS	−0.04, *P* = NS	0.20, *P* = NS	−0.12, *P* = NS	−0.23, *P* = NS
SPARCC MRI SIJ inflammation score	0.12, *P* = NS	0.12, *P* = NS		**−0.28, *P *** **= 0.041**	**0.29, *P *** **= 0.032**	**−0.34, *P *** **= 0.011**	−0.02, *P* = NS
SSS for fat metaplasia	**−0.27, *P *** **= 0.023**	−0.07, *P* = NS	**−0.40, *P *** **= 0.001**		−0.08, *P* = NS	**0.31, *P *** **= 0.0097**	0.21, *P* = 0.092
SSS for erosion	0.023, *P* = NS	−0.23, *P* = NS	**0.49, *P *** **< 0.0001**	**−0.54, *P *** **< 0.0001**		**−0.39, *P *** **= 0.0011**	**−0.33, *P *** **= 0.0058**
SSS for backfill	−0.11, *P* = NS	0.04, *P* = NS	**−0.39, *P *** **= 0.001**	**0.30, *P *** **= 0.008**	**−0.46 < 0.0001**		−0.02, *P* = NS
SSS for ankylosis	0.10, *P* = NS	0.07, *P* = NS	−0.09, *P* = NS	**0.35, *P *** **= 0.0018**	**−0.33, *P *** **= 0.0027**	0.06, *P* = NS	

Data presented as Spearman’s rho and P values. Data in bold are statistically significant. ASDAS, Ankylosing Spondylitis Disease Activity Score; CRP, C-reactive protein; MRI, magnetic resonance imaging; NS, not significant; SIJ, sacroiliac joint; SPARCC, Spondyloarthritis Research Consortium of Canada; SSS, Sacroiliac Joint Structural Score; TNFα, tumor necrosis factor alpha.

### Univariate analyses

#### Change in erosion

Neither gender nor B27 status was associated with change in erosion. For disease activity measures, the baseline ASDAS (β = −0.40, *P* = 0.052) and baseline and 2-year change in SPARCC MRI SIJ inflammation score (β = −0.12, *P* = 0.002 and β = 0.17, *P* < 0.0001, respectively) were associated with change in erosion. For structural damage parameters, the baseline SSS for ankylosis (β = 0.088, *P* = 0.002) and baseline SSS for erosion (β = −0.39, *P* < 0.0001) were associated with change in erosion. Significant interaction between baseline variables and treatment for change in erosion was only evident for baseline SSS for erosion.

#### Change in fat metaplasia

Neither gender nor B27 status was associated with change in fat metaplasia. For disease activity measures, the baseline and 2-year change in ASDAS (β = −0.36, *P* = 0.009 and β = −0.36, *P* = 0.009, respectively), the baseline and 2-year change in CRP (β = 0.025, *P* = 0.014 and β = −0.027, *P* = 0.006, respectively), and the baseline and 2-year change in SPARCC MRI SIJ inflammation score (β = 0.059, *P* = 0.019 and β = −0.08, *P* = 0.005, respectively) were associated with change in fat metaplasia. For baseline structural damage parameters, the SSS for backfill (β = 0.076, *P* = 0.057), SSS for erosion (β = 0.11, *P* = 0.006), and SSS for fat metaplasia (β = 0.06, *P* = 0.025) were associated with change in fat metaplasia. There were no significant interactions between baseline variables and treatment for change in fat metaplasia.

### Multivariate analyses

Because treatment group differences for change in fat metaplasia SSS and erosion SSS were significant, the effect of treatment on change in these scores was further analyzed in multivariate stepwise regression that included demographic variables as well as the variables that were significant (*P* < 0.10) in univariate analysis.

Significant variables independently associated with change in SSS for erosion were treatment, 2-year change in SPARCC SIJ inflammation score, and baseline SSS for erosion (Table 4). No significant interaction was evident between either treatment and gender or treatment and B27 in regression analyses addressing change in erosion. A significant interaction was observed for treatment and baseline SSS for erosion (β = −0.46, *P* < 0.0001) but not for other baseline SSS. The difference in erosion change scores between the standard and anti-TNFα groups with the same baseline score depends on the baseline SSS for erosion. The higher the baseline SSS for erosion, the greater the expected difference in SSS erosion change scores between the standard and anti-TNFα groups (Figure S1 in Additional file 1). Addition of the interaction term treatment/baseline SSS for erosion to the regression model that included all of the variables cited in Table 4 increased the explained variance in the dependent variable and the adjusted *R*^2^ from 0.44 to 0.52.

**Table 4:** Multivariate stepwise regression analyses of data from 147 patients with axial spondyloarthritis followed over 2 years assessing the associations between treatment and development of erosion or fat metaplasia

	**β coefficient**	**SE**	***t*** **value**	***P*** **value**
TNFα inhibitor treatment	−1.33	0.47	−3.0	0.003
2 year change in SPARCC SIJ inflammation score	0.09	0.036	2.57	0.012
Baseline SSS erosion score	−0.40	0.059	−7.1	<0.0001

Dependent variable: 2-year change in SSS for erosion. Adjusted *R*^2^ = 0.45. Variables not included in the model: age, sex, symptom duration, baseline Ankylosing Spondylitis Disease Activity Score, baseline SPARCC SIJ inflammation score, and baseline SSS for ankylosis. *SE, standard error;* SIJ, sacroiliac joint; SPARCC, Spondyloarthritis Research Consortium of Canada; SSS, Sacroiliac Joint Structural Score; TNFα, tumor necrosis factor alpha.

Significant variables independently associated with change in SSS for fat metaplasia were 2-year change in ASDAS, 2-year change in SPARCC MRI SIJ inflammation score, and baseline SSS for fat metaplasia (Table 5). No significant interaction was evident between treatment and gender, between treatment and B27, or between treatment and baseline SSS in regression analyses addressing change in fat metaplasia (data not shown).

**Table 5:** Multivariate stepwise regression analyses of data from 147 patients with axial spondyloarthritis followed over 2 years assessing the associations between treatment and development of erosion or fat metaplasia

	**β coefficient**	**SE**	***t*** **value**	***P*** **value**
2 year change in ASDAS	−0.43	0.15	−2.83	0.006
2 year change in SPARCC SIJ inflammation score	−0.077	0.032	−2.40	0.019
Baseline SSS score for fat metaplasia	0.085	0.039	2.16	0.034

Dependent variable: 2-year change in SSS for fat metaplasia. Adjusted *R*^2^ = 0.18. Variables not included in the model: age, sex, symptom duration, treatment, baseline ASDAS, baseline and 2-year change in C-reactive protein, baseline SPARCC SIJ inflammation score, baseline SSS for backfill, and baseline SSS for erosion. *ASDAS,* Ankylosing Spondylitis Disease Activity Score; *SE, standard error; SIJ*, sacroiliac joint; *SPARCC*, Spondyloarthritis Research Consortium of Canada; *SSS*, sacroiliac joint structural score.

## Discussion

We have developed a scoring method for structural lesions in the SIJs, which is based on the same scoring principles used in the SPARCC MRI SIJ inflammation scoring method. The approach to the selection of MRI slices is anatomically defined, the majority of the cartilaginous portion of the joint is assessed on consecutive slices in the semi-coronal plane, lesions are scored in SIJ quadrants, and scoring is dichotomous (present/absent), which simplifies assessment and improves reliability. The pathological abnormalities visible on MRI often include mixed lesions with complex anatomical appearances, which may challenge scoring approaches based on estimates for percent volume of the SIJ quadrant. This analysis was aimed at generating preliminary data on discrimination and showed that the SSS method could detect treatment group differences in the magnitude of change over 2 years between patients on standard and TNFα inhibitor therapies. The latter group demonstrated a significantly higher increase in SSS for fat metaplasia as well as significantly greater decrease in SSS for erosion compared with patients on standard therapy. Our data support the hypothesis that resolution of inflammation is associated with reduction in erosion and development of fat metaplasia and that this is more likely to occur following treatment with TNFα inhibitors.

There has been limited study aimed at development and validation of structural lesions in the SIJs using MRI. Erosion has been quantified according to a grading scheme based on the number of erosions per SIJ (1 = 1 to 2 erosions per SIJ, 2 = 3 to 5 erosions per SIJ, 3 = >5 erosions per SIJ) [3]. Reliability for status score was very good but data for change scores were not reported. In a second method, erosion was graded in both cartilaginous and ligamentous compartments and severity was graded according to the extent of subcortical bone affected (0 = no erosion, 1 = <25% erosion, 2 = 25 to 50% erosion, 3 = >50% erosion) [16]. Reliability for status score was good but data for change scores were not reported. Assessment of erosion in the ligamentous compartment may be challenging due to the presence of normal ligamentary insertions associated with irregularity of cortical bone that may simulate the appearance of erosion. The complex anatomy of the joint together with the frequent finding of complex patterns of structural lesions that occur in combination on the same MRI slices may also challenge reliable estimation of extent of involvement that is based on number of erosions per joint or percent of subcortical bone affected.

Our observations indicate that the morphology of erosion may change as inflammation resolves. We have defined erosion as a full thickness breach of cortical bone together with loss of the adjacent bright marrow signal on T1WSE scans, indicating replacement of normal fatty marrow by inflammatory tissue. We previously hypothesized that resolution of inflammation is associated with sclerosis at the edge of the resorbed cavity and infilling of the cavity with tissue that demonstrates an increased signal on T1WSE MRI consistent with fat metaplasia [5]. Backfill is the term we gave to this appearance of new tissue with high signal on T1WSE MRI that is clearly demarcated from adjacent marrow by an irregular dark signal reflecting sclerosis (Figure three in [5]). This evolution of erosion to backfill was evident in one-third of patients in this study cohort after 2 years of follow up, and correlation analysis demonstrated a significant association with resolution of inflammation irrespective of treatment. The complex morphology of backfill requires more calibration than other structural lesions in the SIJ, as shown by the higher relative cutoff value for SDC.

Several reports have shown that fat metaplasia occurs following resolution of inflammation in SpA and several scoring methods have been developed, although none as yet has been validated to show that change scores can be reliably detected. One report assessed fat lesions dichotomously (present/absent) according to SIJ quadrants and a 0 to 8 scoring range based on a global evaluation of the SIJ rather than scoring of SIJ quadrants in individual slices [3]. Development of new lesions correlated with resolution of inflammation and a significant difference was noted between patients on etanercept versus those on salazopyrine as soon as 24 weeks. Reliability for status scores was very good but data for change scores were not reported. Nevertheless, these data are consistent with our data and show that assessment of fat metaplasia may be discriminatory, especially in comparisons that include patients on TNFα inhibitor therapy. A second method grades fat metaplasia in both cartilaginous and ligamentous compartments and grades severity according to the extent of subcortical bone affected (0 = no fat, 1 = <25% fat, 2 = 25 to 50% fat, 3 = >50% fat) [16]. A weighting of 1 is added for fat metaplasia extending ≥1 cm beneath the joint surface. This method has only been validated for reliability of status score, which was reported as good. A limitation of this approach is that fat metaplasia may also occur beyond the subcortical region and its presence in a subcortical region may be less specific for SpA [17].

The primary study limitation is that the data are derived from an observational cohort and not from randomized groups so that patients receiving TNFα inhibitors had more severe disease at baseline. In particular, change in any structural damage parameter may reflect the possibility that there are more severe structural changes at baseline in the TNFα inhibitor group due to confounding by indication. Such patients may therefore be more likely to show further structural changes over time, which may not reflect a treatment effect but a more severe disease phenotype. Multivariate analysis adjusted for extent of structural damage at baseline and demographic variables showed that reduction in inflammation, defined by decreased SPARCC MRI SSS for inflammation, and category of treatment were both independently associated with reduction in erosion score. Change in erosion also depended on the extent of erosion at baseline as defined by the SSS for erosion but there was evidence of a significant interaction with treatment. The most probable interpretation of this finding is that the observed reduction in erosion is more likely in the TNFα inhibitor group the greater the extent of erosion at the start of therapy, and this is associated with its anti-inflammatory effect. Category of treatment did not emerge as a significantly associated variable in multivariate analysis of change in fat metaplasia adjusted for structural damage at baseline and demographic variables. Change in measures of inflammation (ASDAS, SPARCC MRI SIJ inflammation score) and baseline SSS for fat metaplasia were independently associated with 2-year change in fat metaplasia, suggesting that the likelihood of developing fat metaplasia may be more strongly associated with a particular phenotype of disease rather than a specific treatment.

A second major limitation of this study is that MRI assessments were conducted 2 years apart and it is necessary to demonstrate whether change in structural lesions can be reliably demonstrated within shorter time frames that may allow the conduct of randomized studies with feasible sample sizes. Lesion characteristics need to be further defined through comparative studies using computed tomography, and their prognostic significance should be analyzed to determine whether they could be useful surrogate endpoints in trials of disease-modifying therapies.

## Conclusions

We have tested the discriminative metrics of a new scoring methodology for the assessment of structural lesions on MRI in the SIJ of patients with SpA that is based on the same principles as the widely used SPARCC MRI SIJ inflammation score. We show greater fat metaplasia and less erosion over 2 years in patients receiving TNFα inhibitors compared with those on standard therapy. Our prospective data support the hypothesis that both resolution of erosion and development of new fat metaplasia are associated with the resolution of inflammation. However, development of new fat metaplasia may also be more directly associated with a particular phenotype of disease rather than the use of any specific therapeutic agent. Further validation for discriminatory capacity over shorter time frames and for prognostic significance is warranted.

## Abbreviations

ASDAS: Ankylosing Spondylitis Disease Activity Score; CRP: C-reactive protein; MRI: magnetic resonance imaging; SDC: smallest detectable change; SIJ: sacroiliac joint; SpA: spondyloarthritis; SPARCC: Spondyloarthritis Research Consortium of Canada; SSS: Sacroiliac Joint Structural Score; T1WSE: T1-weighted spin echo; TNFα: tumor necrosis factor alpha.

## Competing interests

The authors declare that they have no competing interests.

## Authors’ contributions

SJP and SW contributed to the development of the scoring method, was one of the MRI readers, and contributed to data analysis and drafting of the manuscript. PC, RGL and WPM contributed to the development of the scoring method, data analysis, and drafting of the manuscript. All authors read and approved the final manuscript.

## Supplementary Material

Additional file 1: Figure S1 Depicting predicted erosion change scores for regression equation without baseline SSS for erosion/treatment interaction. The lines are parallel but the intercepts are different. Regardless of the SSS for baseline erosion, the predicted difference in erosion change scores between the treatment group and standard group with the same baseline erosion SSS is −1.32. Click here for file
