# Supplementary material for: Tumor necrosis factor inhibitor therapy but not standard therapy is associated with resolution of erosion in the sacroiliac joints of patients with axial spondyloarthritis
Source: Arthritis Res Ther. 2014 Apr 22;16(2):R100. doi: 10.1186/ar4548 (PMC4060567; doi:10.1186/ar4548)
Supplement: Additional file 1: Figure S1 — Depicting predicted erosion change scores for regression equation without baseline SSS for erosion/treatment interaction. The lines are parallel but the intercepts are different. Regardless of the SSS for baseline erosion, the predicted difference in erosion change scores between the treatment group and standard group with the same baseline erosion SSS is −1.32. [file ar4548-S1.docx]

**Supplementary figure**. Graph depicting predicted erosion change scores for regression equation without baseline SSS erosion score/treatment interaction. The lines are parallel but the intercepts are different. Regardless of the SSS baseline erosion SSS score, the predicted difference in erosion change scores between the treatment group and standard group with the same baseline erosion SSS score is -1.32.

Graph 2 with the interaction term shows that the difference in erosion change scores between the standard and anti-TNF groups with the same baseline score depends on the baseline Erosion SSS score. The higher the BL Erosion SSS score, the greater the expected difference in erosion change scores between the standard and anti-TNF groups.
